# Shared cognitive mechanisms of hypnotizability with executive functioning and information salience

**DOI:** 10.1038/s41598-021-84954-8

**Published:** 2021-03-11

**Authors:** Afik Faerman, David Spiegel

**Affiliations:** 1 Department of Psychology, Palo Alto University, Palo Alto, CA USA; 2 Psychiatry and Behavioral Sciences, Stanford University, Palo Alto, CA USA

**Keywords:** Psychology, Human behaviour

## Abstract

In recent years, evidence linked hypnotizability to the executive control and information salience networks, brain structures that play a role in cognitive conflict resolution and perseveration (insisting on applying a previously learned logical rule on a new set). Despite the growing body of neuroimaging evidence, the cognitive phenotype of hypnotizability is not well understood. We hypothesized that higher hypnotizability would correspond to lower perseveration and set-shifting. Seventy-two healthy adults were tested for hypnotizability and executive functions (perseveration and set-shifting). Multiple regression analyses were performed to test the relationship between hypnotizability and perseveration and set-shifting. Higher hypnotizability was associated with lower perseveration after accounting for age and education. Hypnotizability significantly predicted perseveration but not set-shifting. Our results indicate an inverse relationship between trait hypnotizability and perseveration, an executive function that utilizes regions of both the executive control and the salience systems. This suggests that hypnotizability may share a common cognitive mechanism with error evaluation and implementation of logical rules.

## Introduction

Ralph Waldo Emerson famously opined that “a foolish consistency is the hobgoblin of little minds, adored by little statesmen and philosophers and divines”^1^. The ability to counter such mundane consistencies would, from his perspective, be a positive attribute—a form of cognitive flexibility. Hypnosis, the oldest psychotherapeutic technique in Western medicine^2^, is often disparaged as susceptibility to the imposition of mental frameworks by others, yet actually represents a robust capacity to adopt novel mental sets. If so, current neuropsychological science should provide evidence that the capacity to experience hypnosis is linked to cognitive flexibility.

Hypnotizability is a stable^3^ multifaceted trait representing one’s ability to experience physiological, sensory, behavioral, and emotional phenomena in response to suggestions given during hypnosis^4^. Recent evidence from neuroimaging studies revealed relationships between hypnotizability and brain regions central to executive functions and information processing^5^. For example, hypnotizability, outside hypnosis, was positively associated with functional connectivity between central nodes of the executive control network (left dorsolateral prefrontal cortex; lDLPFC) and the salience network (mainly the dorsal region of the anterior cingulate cortex; dACC), which play a central role in monitoring for conflict in contextual processing of information^6^, among its involvement in a diverse array of cognitive functions. Both the prefrontal cortex (PFC)^7–10^ and the anterior cingulate cortex (ACC)^7,8,11–16^ show altered activation that is related to high hypnotizability during hypnotic analgesia (hypnosis-related modulation of pain perception), Stroop interference, and during the resting hypnotic state. Hypnosis studies using electroencephalographic (EEG) correlates of hypnotizability further support differential functional organization of neural networks in highly hypnotizable compared to low hypnotizable individuals^17^, including disruptions in frontoparietal network connectivity^18^. Furthermore, outside the context of hypnosis, high hypnotizability is associated with greater recruitment of the right inferior frontal gyrus (rIFG) and reduced recruitment of the ACC and intraparietal sulcus (IPS) during incongruent trials of selective attention tasks^16^. Cojan et al.^16^ interpreted these results by arguing that high hypnotizability is related to increased exertion of executive control when confronted by cognitive conflict. Moreover, the authors noted that high hypnotizability was associated with greater connectivity between the rIFG and the default mode network and suggested higher flexibility in attentional abilities. Conversely, when facing distracters in tasks of selective attention, high hypnotizability was related to less recruitment of parietal and anterior cingulate regions conditions, reflecting attenuated detection and resolution of attentional conflicts^16^. Interestingly, decreased activity in the dACC in high hypnotizable individuals was also observed during hypnosis^19^ and when using hypnosis for the purpose of interfering with the Stroop effect^9^. These findings are complemented by our team’s recent finding that higher hypnotizability is associated with a greater concentration of GABA, an inhibitory neurotransmitter, in the ACC^20^. Overall, the PFC (and the DLPFC in particular), the ACC (and the dACC in particular), and potentially the rIFG appear to be neurocognitively involved in hypnotizability, and their activity might represent neurophysiological markers of hypnotizability.

Hypnotizability has been previously proposed to represent an alteration in attentional-executive systems that involve frontal and limbic brain structures^21^ or as an attention-focused preparatory response set that allows automatic activation of behavioral responses to suggestion^22,23^. Greater coordination between the executive control and salience networks would be expected to enhance the intensity of focal attention seen during hypnosis and has been observed using fMRI in the form of higher functional connectivity between the left DLPFC and the dACC among high as compared with low hypnotizable individuals^6^. Both Crawford^24,25^ and Gruzelier^25,26^ hypothesized that hypnotic responsiveness relates to inhibitory processes of attentional and information processing systems. While higher hypnotizability is related to faster reaction time on simple response tasks^27,28^, the inhibitory component of hypnotizability manifests as slower reaction time on more complex attentional tasks with inhibitory demands^27^. Consistently, comparing the performance of high versus low hypnotizable individuals on the Attentional Blink task, Castellani et al.^29^ noted that cognitive performance in high hypnotizable individuals might be more sensitive to time constraints and increased cognitive complexity. However, the relationship between hypnotizability and cognitive inhibition is not entirely consistent. For example, Dienes et al.^30^ found no correlations between hypnotizability and cognitive inhibition, on three independent measures, in a large sample of 180 participants. Thus, it is possible that it was not the inhibitory component of the mentioned tasks that drives the observed relationships. High hypnotizability was also associated with increased automaticity in verbal processing, manifested as a greater slowing in reaction time on incongruent trials of the Stroop test^9,31^ and faster encoding time on the word-stem completion task^32^, outside the context of hypnosis. Notably, some studies found no evidence of reaction-time based performance differences, on various aspects of attention, between individuals with high or low hypnotizability outside hypnosis^33,34^. Others found that when hypnotized subjects were instructed that the color words were written in a foreign language that they did not understand, subjects did not demonstrate Stroop interference^35^. This effect has been associated with reduced peripheral attention as measured by visual event-related potentials^36^. Overall, when operationalizing waking cognitive abilities vis-à-vis processing speed and reaction time, it appears that high hypnotizability might provide a small advantage in simple attention tasks and variable delays as task complexity and demands increase.

While the cognitive mechanisms of trait hypnotizability, outside the context of hypnosis, have been studied mainly through measures of timed performance, there is a paucity of evidence on untimed cognitive tasks. For example, in a small study, Aikins and Ray^37^ found that individuals with high hypnotizability completed the Wisconsin Card Sorting Test in fewer trials than those with low hypnotizability. Khodaverdi-Khani and Laurence^38^ found hypnotizability to correlate negatively with working memory (Digit Span Backwards) but not simple attention (Digit Span Forward) performance, but noted that high hypnotizable individuals scored significantly lower than low hypnotizable individuals on both tasks. They complemented their findings by including the N-back test, showing that high hypnotizable individuals were able to significantly improve their working memory performance over time, while low hypnotizable’s performance decreased^38^. They interpreted this finding as a potential ability of high hypnotizables to automate the task and become maintain efficiency over time^38^. Farvolden and Woody^39^ found that highly hypnotizable individuals perform worse on verbal memory tasks with greater frontal demands and largely similar on tasks with relatively lower frontal demands in comparison to low hypnotizable individuals. Furthermore, they noted that participants’ performances across free-recall, proactive interference, and source amnesia tasks were evident both outside and within the context of hypnosis^39^, suggesting that some relationships between cognitive performance and hypnotizability are not dependent on formal hypnotic induction and likely represent frontal cognitive mechanisms underlying hypnotizability. Terhune et al.^40^ further elaborated on Farvolden and Woody’s^39^ findings and found that only those high in hypnotizability that are high in dissociative phenomenology perform lower on the same verbal memory task, as well as on a measure of working memory, compared to both low-dissociative high hypnotizable and low hypnotizable individuals. Low-dissociative high hypnotizable individuals did not differ in their performance from low hypnotizables on either task. It is important to note that while verbal memory performance was not measured via reaction time, both Farvolden and Woody’s^39^ and Terhune et al.^40^ used a modified version of the Auditory Verbal Learning Test (AVLT), which limited participants to 30 s recall windows. As such, processing speed differences might have had an impact on their findings. Overall, existing evidence largely supports the involvement of hypnotizability in frontal functions, outside the context of hypnosis^5^. However, performance on untimed measures of executive functioning and other aspects of frontal activity has not been covered thoroughly in existing literature. For more comprehensive review of executive functioning and hypnotizability, see Parris^5^.

As noted earlier, the main characteristic of high hypnotizability is the heightened tendency to accept hypnotic suggestions as salient and “true” and successfully experience the suggested phenomena. Conceptually, such an affinity for suggestions can be partially modulated through reduced criticality and increased tendency to accept new logical rules, consistent with reduced activity in the salience network, particularly in conflict-detecting functions of the dACC^19,20^. Put differently, individuals high in hypnotizability might transition more easily between previously learned rules and a novel suggested rules. For example, when one is suggested that her hand becomes light and will float upwards, a person with low levels of hypnotizability is likely to experience dissonance between her existing cognitive schemas and the suggestion, might evaluate it critically as an unsubstantiated claim and, consequently, is more likely to reject the suggestion (whether consciously or less so). Conversely, a highly hypnotizable person might have a relative attenuation of the cognitive dissonance and critical evaluative processes when interacting with the suggestion and is, thus, more likely to successfully experience the suggested phenomena. Such attenuation of critical evaluative processes might also manifest in easier shifts between logical rules in people who are highly hypnotizable. This hypothesis is also consistent with previous interpretations that highly hypnotizable individuals implement strategic adjustments better than those with low hypnotizability^31^. The insistence on applying previously learned logical rules when presented with an alternative rule has been thoroughly studied in neuropsychology under the term “perseveration.”^41^ While neuropsychological studies of perseveration were done mainly in the context of cognitive impairment (e.g., following brain injury or neurodegenerative disease), some degrees of perseveration are normal and indicative of active conflict management processes. In the context of hypnotizability, when excluding the effects of time constraints, it is possible that higher hypnotizability relates to less perseveration (i.e., lower likelihood of insisting on applying a previously learned logical rule given an alterative rule and, thus, a greater likelihood to accept hypnotic suggestions).

**Figure 1:**
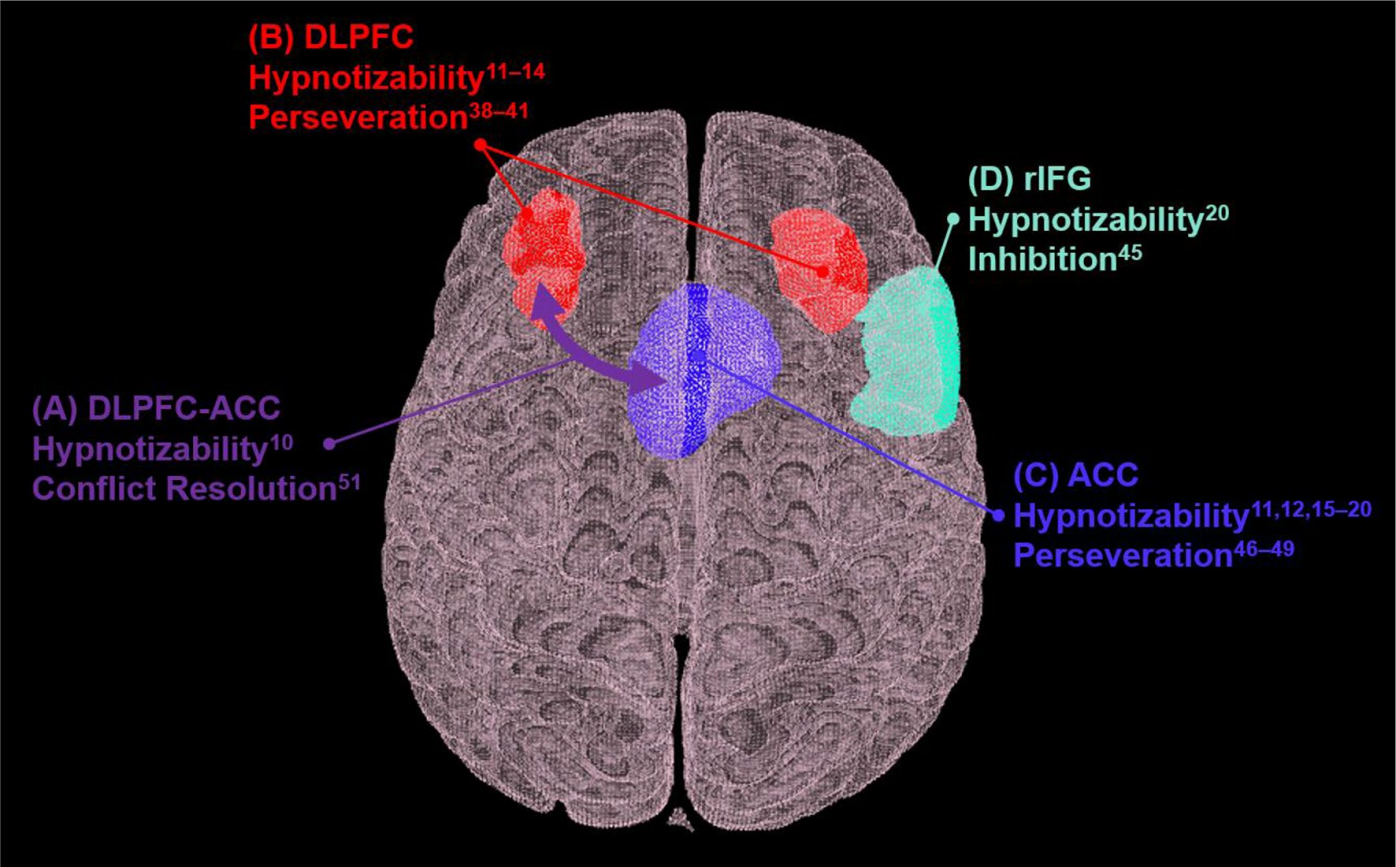
Summary of brain regions of interest. Hypnotizability: (**A**) Hypnotizability is associated with increased functional connectivity between left DLPFC and the dACC ^6^; High hypnotizability is related to altered activity in the (**B**) DLPFC^7–10^ and the (**C**) ACC ^7,8,11–16^, and is associated with (**D**) greater recruitment of the rIFG during incongruent trials of selective attention tasks ^16^. Perseveration: (**A**) The interplay between the DLPFC and ACC plays a central role in the processing of cognitive conflicts^54^; The (**B**) PFC is related to perseveration in both humans ^41–44^ and nonhuman primates ^45–47^; The (**C**) ACC is also associated with perseveration in both human ^49–52^ and animal models ^53^; (**D**) The rIFC (includes the rIFG) is a central component in the inhibition of executive control ^48^; Lower performance on the WCST is linked to impairments in the (**B**) PFC, (**C**) ACC, and (**D**) IFC ^63–65^. ACC = anterior cingulate cortex; DLPFC = dorsolateral prefrontal cortex; PFC = prefrontal cortex; rIFC = right inferior frontal cortex; rIFG = right inferior frontal gyrus; WCST = Wisconsin Card Sorting Test.

Neuroimaging correlates of hypnotizability suggest additional support to a potential shared cognitive mechanism with perseveration. The prefrontal cortex, a region involved in executive control, cognitive flexibility, and monitoring performance^6^, has been long linked with perseveration in both humans^41–44^ and nonhuman primates^45–47^. Furthermore, the right inferior frontal cortex (IFC) is a central component in the inhibition of executive control^48^. Moreover, evidence points to a relationship between the ACC and perseveration in both human^49–52^ and animal models^53^. Mansouri et al.^54^ hypothesized that the interplay between the ACC and the DLPFC could be conceptualized by highlighting that the ACC plays a role in conflict detection (or context-driven uncertainty stimuli) and the DLPFC is involved in response selection within the task context^54^. Following the evidence that the PFC, ACC, and IFG are involved in hypnotizability, it is plausible that hypnotizability shares common cognitive mechanisms with the aspects of executive functions that are carried out in those regions. A visual summary of the brain regions discussed and their relevance to hypnotizability and the aforementioned cognitive functions is presented in Fig. 1.

In the current study, we hypothesized that higher levels of hypnotizability would relate to (a) less perseveration, (b) shorter reaction times on simple attentional tasks^27^, and (c) increased slowing effects as cognitive complexity increases^27,29^ (e.g., cognitive set-shifting). In particular, we propose a shared cognitive mechanism between hypnotizability and both perseveration and cognitive set-shifting.

## Methods

### Participants

**Figure 2:**
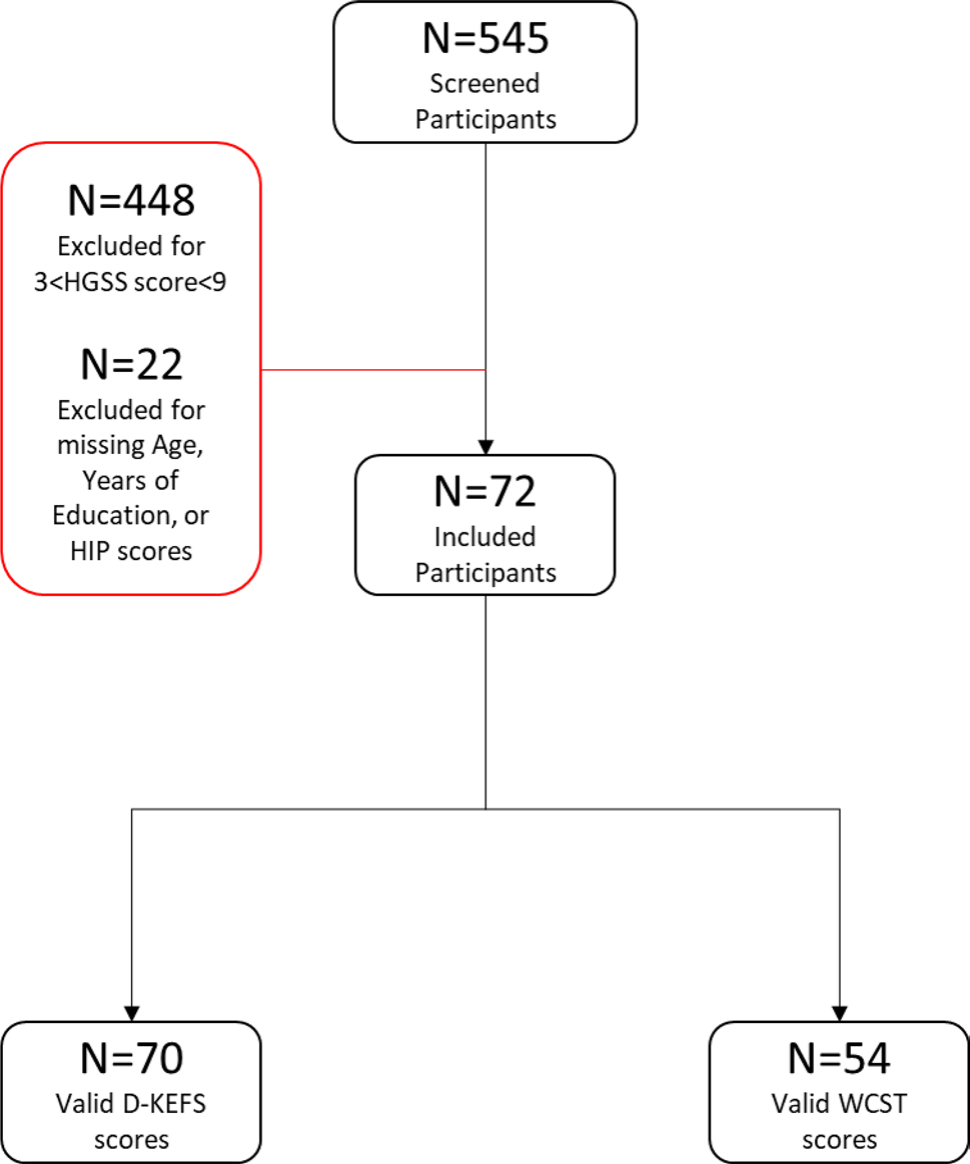
Consort diagram of included and excluded participants. D-KEFS = Delis-Kaplan Executive Functions System; HGSHS:A = Harvard Group Scale of Hypnotic Susceptibility; WCST = Wisconsin Card Sorting Test.

Study protocols were approved by the Stanford University Institutional Review Board (IRB) and were carried out in accordance with the approved guidelines. Participants were adults, 18 years of age or older, who were recruited in university settings for a study exploring functional brain activity and connectivity associated with hypnosis^19^. Participants provided were taking no medications at the time of recruitment and were free of psychiatric, neurologic, or substance use disorders. All participants provided informed consent. To recruit a sample that included individuals who were low or highly hypnotizable, 545 prospective participants were administered the Harvard Group Scale of Hypnotic Susceptibility, Form A (HGSHS:A^55^). Individuals with high hypnotizability (i.e., HGSHS:A scores of 9–12; 61% of participants) and low hypnotizability (i.e., HGSHS:A scores of 0–3; 39% of participants) were recruited. Experimenters were blind to participants’ hypnotizability scores and classifications at all phases of the data collection, coding, and analysis. Seventy-two participants (59% female, mean age 25. 5 ± 11.6) were included in the current study for the purpose of reporting previously unexamined neuropsychological performance data (see Fig. 2 for an inclusion consort diagram). Participants’ demographic information is presented in Table 1.

**Table 1:** Demographics.

	*Subsample 1* *N* = 54	*Subsample 2* N = 70
Age	24.0 ± 10.1	25. 6 ± 11.8
Gender	59% female	59% female
Race/ethnicity*		
White/Caucasian	45.8%	51.4%
African American / Black	8.5%	7.1%
Asian	18.6%	18.6%
Hispanic	6.8%	8.6%
Multiracial	6.8%	8.6%
Other	5.1%	4.3%
Years of Education	12.9 ± 6.1	13.7 ± 5.7
**HIP**		
Mean	8.4 ± 2.8	8.3 ± 3.0
Range	0–12	0–12
Skewness	− 1.58 ± .32	− 1.39 ± .29
Kurtosis	1.89 ± .63	1.22 ± .57
**WCST**		
Number of Trials	88.6 ± 17.2	**
Total Errors	104.9 ± 14.9	**
Perseverative Responses	108.8 ± 19.2	**
Perseverative Errors	108.2 ± 18.8	**
Nonperseverative Errors	103.2 ± 14.9	**
**D-KEFS TMT**		
Condition 1	**	11.4 ± 2.0
Condition 2	**	11.4 ± 2.1
Condition 3	**	11.5 ± 2.3
Condition 4	**	11.3 ± 2.5
Condition 5	**	11.4 ± 2.0

*D-KEFS* Delis–Kaplan Executive Function System, *HIP* Hypnotic Induction Profile, *TMT* Trail Making Test, *WCST* Wisconsin Card Sorting Test.

*Five participants did not check any race/ethnicity.

**Was not analyzed in this subsample.

### Measures

#### Hypnotic induction profile (HIP) and the Harvard group scale of hypnotic susceptibility, form A (HGSHS:A)

The HIP^56–58^ is a standardized evaluation of trait hypnotizability, with a reliability coefficient (ICC) of 0.75^59^. Assessment of hypnotizability via the HIP is done by clinician rating of participants’ degree of responsiveness to six hypnotic phenomena: ideomotor response (signaled arm levitation), subjective sense of arm levitation, relative dissociation of the arm, relative nonvolition of the arm, the reversal of the suggested phenomena, and posthypnotic amnesia. The HIP has two validated scoring systems, a 0–10 score that has been thoroughly used in research and clinical practice over the years^56–58^ and a recently validated 0–12 score that includes posthypnotic amnesia^60^. The current study utilized the latter updated scoring system.

The HGSHS:A, is a measure of susceptibility to hypnotic responsiveness was used in the initial prescreening of the original study^19^ to identify individuals who are more likely to be highly or lowly hypnotizable. The HGSHS:A utilizes a hypnotic induction followed by 12 suggestions for ideomotor responses, auditory hallucination, and posthypnotic amnesia, and estimates hypnotic susceptibility on a scale of 0–12 with a reliability coefficient (Kuder-Richardson) of 0.80^55^. Contrary to the one-on-one clinician-administered HIP, the HGSHS:A is a group assessment and relies on examinees’ self-report of their subjective reports of successfully experiencing the suggested phenomena, rather than behaviorally measured by a clinician. Being a group measure, the HGSHS:A has been used as an initial screening of participants. Participants who scored high (HGSHS:A ≥ 9) or low (HGSHS:A ≤ 3) were enrolled in the study^19^ and were evaluated for hypnotizability using the HIP.

#### The Wisconsin card sorting test (WCST)

The WCST is an untimed neuropsychological tool to assess executive functions, mainly the ability to maintain and shift cognitive sets, inhibit control on attention, and form abstract concepts^61,62^. During the test, the examinee is presented with four stimulus cards and are asked to match cards in a given deck to one of the stimulus cards. While examinees do not receive instructions about how to match the cards, they are given feedback on whether each match is right or wrong. The sorting rule changes without warning after a certain number of successful rule matching. Examinees match cards for 128 trials or until successfully completing six categories. The WCST has been robustly linked to frontal and prefrontal functions, with neural correlates in the PFC, ACC, and IFC^63–65^. Lesion-mapping studies show that while the initial categorization rule is often acquired by most, patients with prefrontal damage often struggle with learning and applying a new rule and inhibiting the old one despite corrective feedback^62^.

Among the different scores available in the WCST, in the current study, we used the standard scores (mean ± SD = 100 ± 15) of total errors (i.e., mismatch of the card to the target stimulus), nonperseverative errors (i.e., number of incorrect responses that do not fit the previous matching rule), perseverative errors (i.e., the number of incorrect responses that would have been correct following the previous matching rule), perseverative responses (i.e., the number of responses that would have been correct following the previous matching rule), and number of trials until completion. Higher standard scores reflect better performance (e.g., fewer errors). See Table 1 for means.

#### The Delis-Kaplan executive function system (D-KEFS) trail-making test (TMT)

The TMT is a timed neuropsychological tool to assess visual, psychomotor, and executive functions, mainly the ability to shift cognitive sets on a visuomotor sequencing task^62,66^. Examinees are asked to visually scan the target stimuli and follow five different conditions: (1) marking a target stimulus among letter and number distracters (i.e., Visual Scanning), (2) connecting numbers in ascending order among letter distracters (i.e., Number Sequencing), (3) connecting letters in alphabetical order among number distracters (i.e., Letter Sequencing), (4) alternating between numbers to letters in ascending and alphabetical order (i.e., Number-Letter Sequencing), and (5) drawing over a dotted line (i.e., Motor Speed). While conditions 1, 2, 3, and 5 rely more on basic attentional and motor abilities and pose relatively low cognitive demands (i.e., target identification and maintaining set), the fourth condition requires examinees to inhibit and shift sets (number-letter). The different conditions contextualize the performance on the fourth trial, as they represent the basic requirements to perform on it (with the exception of set-shifting). Several large-scale neuronal networks participate in TMT performance, mostly of prefrontal and parietal regions^67^.

The main performance variables of the TMT are completion times of each of the five conditions. In the current study, we used the scaled scores (mean ± SD = 10 ± 3) of condition completion times. Higher scaled scores reflect better performance (i.e., faster completion times). See Table 1 for means. Although the TMT is less sensitive to perseverative erroring, perseverating will result in slower completion time.

### Statistical analyses

Two-tailed t-tests were used to examine demographic differences between the two subsamples, and neuropsychological performances and hypnotizability were tested for gender differences. Multiple hierarchical regression analyses (two-tailed) were performed using WCST and TMT standard scores as dependent variables and HIP total score as the independent variable. Significant relationships were also tested using age and years of education as covariates.

## Results

The hypotheses were tested using previously unexamined neuropsychological performance data collected as a secondary measure for a study of functional neuroimaging correlates of hypnosis^19^. As neuropsychological data were collected in some, but not all participants, valid test data were available for 54 participants for WCST scores and for 70 participants for TMT scores. As most but not all participants had both WCST and TMT test data, we decided to treat the two groups as subsamples (i.e., subsample 1 with WCST and subsample 2 with TMT data). There were no significant demographic differences between the included and excluded participants in both subsamples. Additionally, no significant differences in neuropsychological performance or hypnotizability have been observed between males and females (all *p* values ≥ 0.168). Although all participants were characterized by either low (39%) or high (61%) hypnotizability based on the HGSHS:A, the HIP confirmed only 64% as high- and 9% as low hypnotizable (i.e., HIP induction scores of 9–12 and 0–3, respectively), leaving 26% as medium hypnotizable. Such dissonance between the low hypnotizable rating might stem from the self- vs. observer-report bias, previously reported in the HGSHS:A^68^. Consistent with previous findings regarding the stability of trait hypnotizability over time^3^, there was no significant correlation between hypnotizability and age (*r* = 0.084, *p* = 0.481). The results of all linear regression models predicting neuropsychological performance by hypnotizability are presented in Table 2.

**Table 2:** Results of linear regression models predicting standardized neuropsychological scores by HIP scores.

Model	*F*	*R*^2^	*B*	*CI*	*β*	*p*
WCST total errors	$F_{{\left( {1, 53} \right)}} =$ 1.510	.028	.901	[− .570, 2.373]	.168	.225
WCST nonperseverative errors	$F_{{\left( {1, 53} \right)}} =$.113	.002	.249	[− 1.238, 1.737]	.047	.738
WCST perseverative errors	$F_{{\left( {1, 53} \right)}} =$ 5.028	.088	2.010	[.211, 3.808]	.297	**.029**
WCST perseverative responses	$F_{{\left( {1, 53} \right)}} =$ 5.277	.092	2.092	[.264, 3.919]	.304	**.026**
WCST number of trials	$F_{{\left( {1, 53} \right)}} =$.192	.004	.376	[− 1.348, 2.100]	.060	.663
TMT 1 Visual Scanning	$F_{{\left( {1, 68} \right)}} =$ 2.380	.034	− .126	[− .288, .037]	− .184	.128
TMT 2 number sequencing	$F_{{\left( {1, 68} \right)}} =$ 7.390	.098	− .223	[− .386, − .059]	− .313	**.008**
TMT 3 letter sequencing	$F_{{\left( {1, 68} \right)}} =$ 1.530	.022	− .116	[− .302, .071]	− .148	.220
TMT 4 letter-number sequencing	$F_{{\left( {1, 68} \right)}} =$ 3.120	.044	− .177	[− .376, .023]	− .209	.082
TMT 5 motor speed	$F_{{\left( {1, 68} \right)}} =$ 2.239	.032	− .120	[− .280, .040]	− .179	.139
TMT Delta 4 versus 1	$F_{{\left( {1, 68} \right)}} =$.218	.003	− .051	[− .269, .167]	− .056	.642
TMT Delta 4 versus 2	$F_{{\left( {1, 68} \right)}} =$.242	.004	.046	[− .140, .232]	.060	.624
TMT Delta 4 versus 3	$F_{{\left( {1, 68} \right)}} =$.388	.006	− .061	[− .257, .135]	− .075	.535

*HIP* Hypnotic Induction Profile, *TMT* Trail Making Test, from the Delis–Kaplan Executive Functioning System, *WCST* Wisconsin Card Sorting Test.

**Figure 3:**
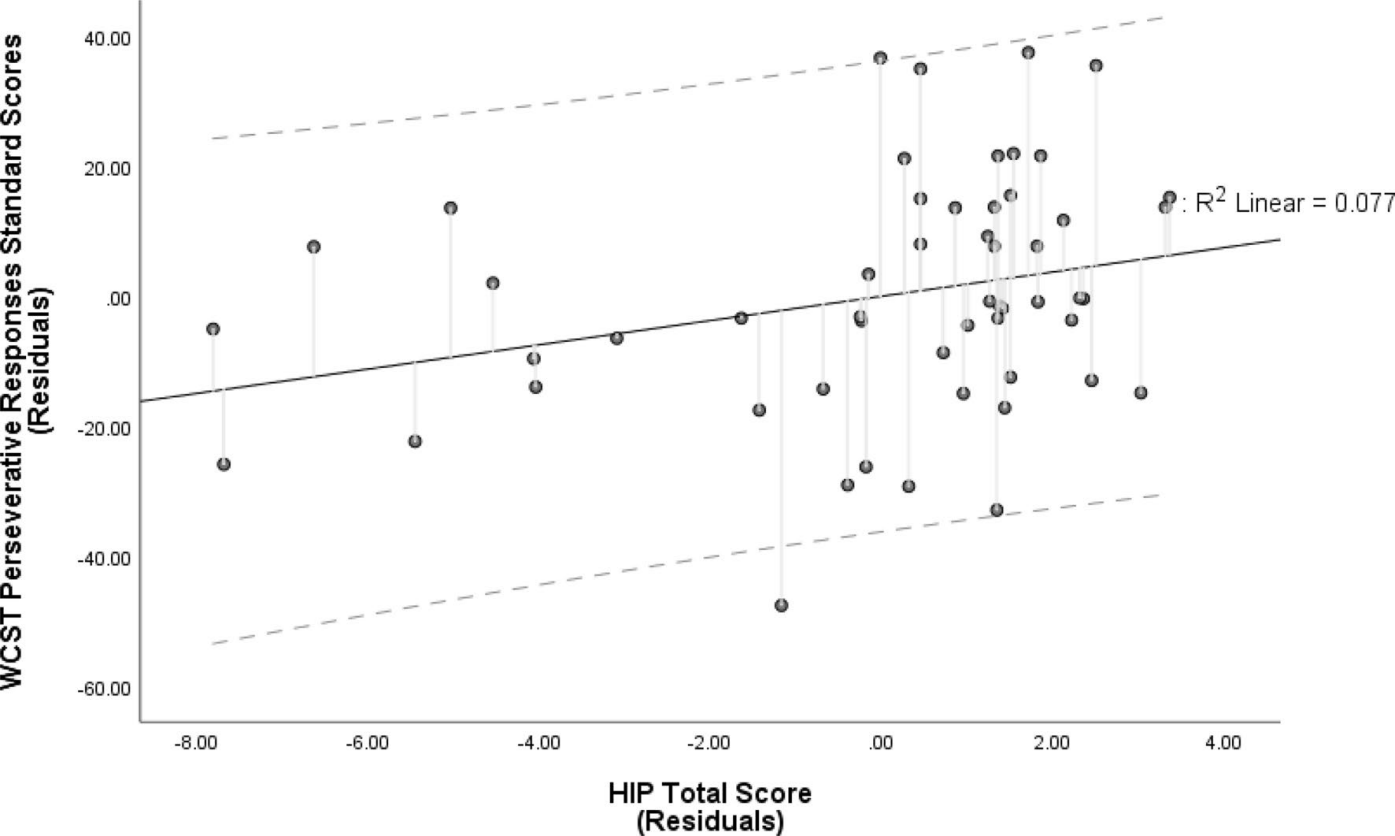
Partial regression plot of the model predicting Perseverative Responses Standard Scores on the Wisconsin Card Sorting Test (WCST) by Hypnotic Induction Profile (HIP) Total Score. The relationship between WCST Perseverative Responses and the HIP total score, after controlling for age and years of education. HIP scores were the only significant predictor of Perseverative Responses Standard Score (higher Standard Scores reflect less perseverative responding). The broken lines represent 95% prediction intervals.

HIP scores significantly predicted the WCST Perseverative Responses standard score (see Table 2), in that higher hypnotizability predicted fewer perseverative responses. The model remained significant after accounting for age and education (*R*^2^ = 0.146, $F_{{\left( {3, 50} \right)}}$ = 2.83, *p* = 0.047), with a medium effect ($f^{2}$ = 0.171) and HIP score as the sole significant predictor (HIP: *B* = 1.868, CI [0.025, 3.711], $t_{{\left( {50} \right)}}$ = 2.036, *β* = 0.271, semi-partial *r* = 0.266, *p* = 0.047; age: *B* = − 0.405, CI [− 0.927, 0.117], $t_{{\left( {50} \right)}}$ = − 1.560, *β* = − 0.213, semi-partial *r* = − 0.204, *p* = 0.125; education: *B* = 0.179, CI [− 0.668, 1.026], $t_{{\left( {50} \right)}}$ = 0.424, *β* = 0.057, semi-partial *r* = 0.055, *p* = 0.673; see Fig. 3). Multicollinearity was not a concern for any of the predictors (all *Tolerance* values ≥ 0.913 and all *VIF* values ≤ 1.095), and the data met assumptions of independent errors (*Durbin-Watson* = 1.95) and of non-zero variances (*Variance* values: WCST = 342.7; HIP = 9.2; Education = 30.4; Age = 186.2). Both the normal P-P plot of standardized residuals and the histogram of standardized residuals and indicated that the data contained largely normally distributed errors.

**Figure 4:**
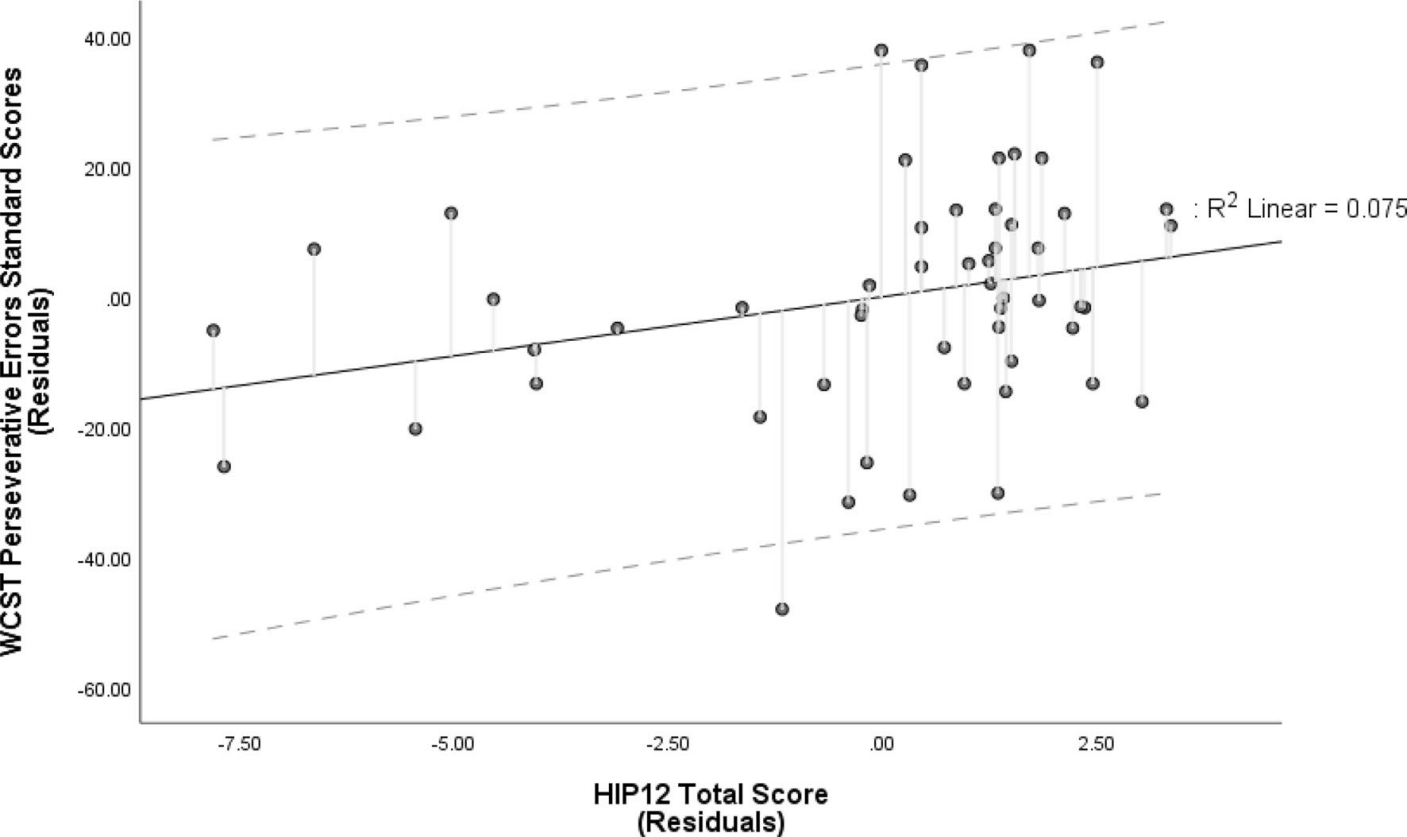
Partial regression plot of the model predicting Perseverative Errors Standard Scores on the Wisconsin Card Sorting Test (WCST) by Hypnotic Induction Profile (HIP) Total Score. The relationship between WCST Perseverative Errors and the HIP total score, after controlling for age and years of education. HIP scores were the only significant predictor of Perseverative Errors Standard Score (higher Standard Scores reflect less perseverative erroring). The broken lines represent 95% prediction intervals.

HIP scores also significantly predicted WCST Perseverative Errors standard scores (see Table 2), in that higher hypnotizability predicted fewer perseverative errors. However, after accounting for age and education, the model showed a non-significant trend in predicting WCST Perseverative Errors (*R*^2^ = 0.137, $F_{{\left( {3, 50} \right)}}$ = 2.65, *p* = 0.059) with HIP score as the sole significant predictor (HIP: *B* = 1.819, CI [0.000, 3.638], $t_{{\left( {50} \right)}}$ = 2.009, *β* = 0.269, semi-partial *r* = 0.264, *p* = 0.050; age: *B* = − 0.361, CI [− 0.876, 0.154], $t_{{\left( {50} \right)}}$ = − 1.409, *β* = − 0.194, semi-partial *r* = − 0.185, *p* = 0.165; education: *B* = 0.231, CI [− 0.605, 1.067], $t_{{\left( {50} \right)}}$ = 0.555, *β* = 0.075, semi-partial *r* = 0.073, *p* = 0.581; see Fig. 4). Neither WCST Total Errors, nor Non-Perseverative Errors or number of trials were significantly predicted by the model (see Table 2). WCST Perseverative Responses and Perseverative Errors correlated significantly (*r* = 0.993, *p* < 0.001) and, thus, their observed relationships with hypnotizability are likely not independent.

**Figure 5:**
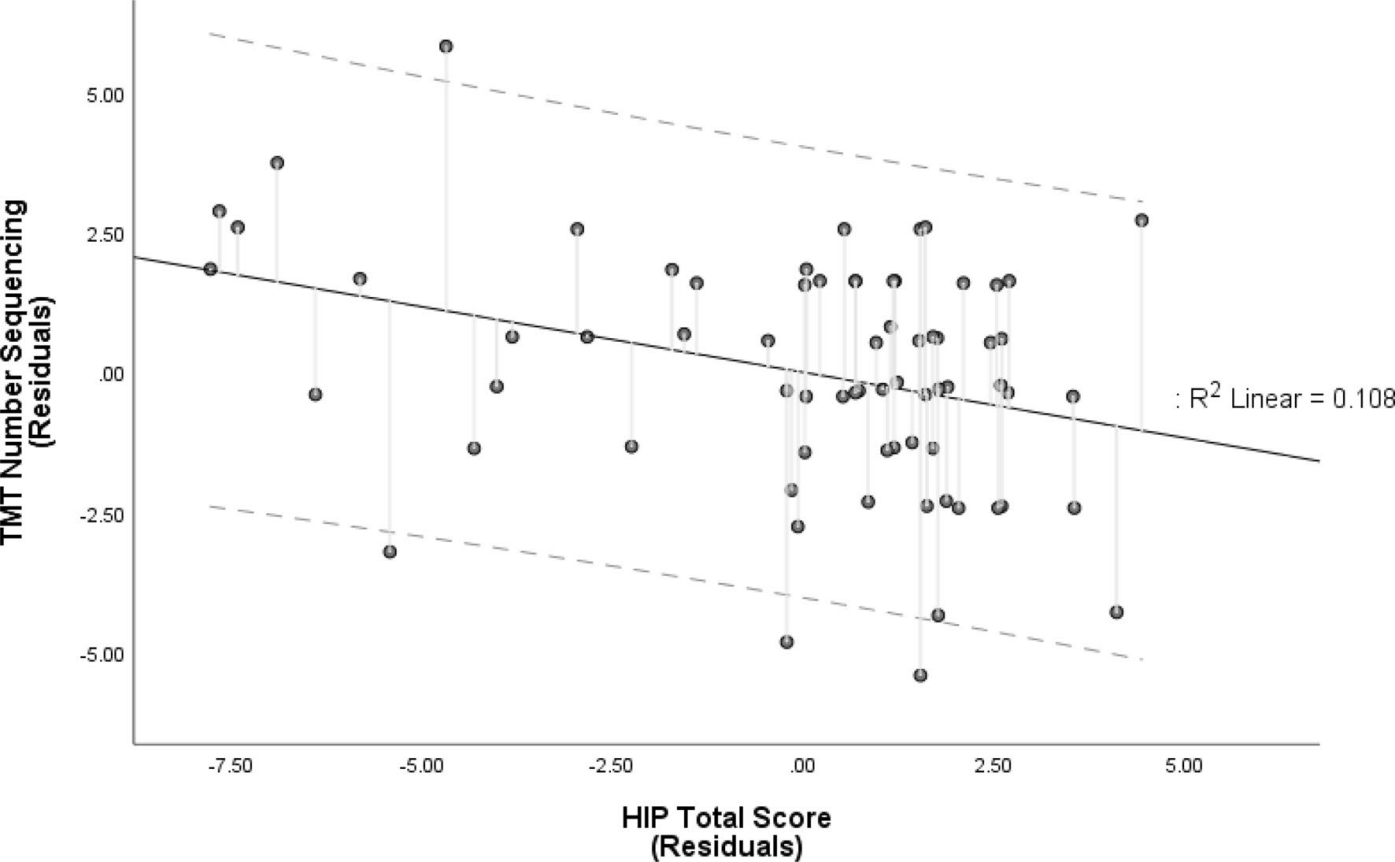
Partial regression plot of the model predicting Trail-Making Test (TMT) Number Sequencing Scaled Scores by Hypnotic Induction Profile (HIP) Total Score. The relationship between TMT Number Sequencing and the HIP total score, after controlling for age and years of education. HIP scores were the only significant predictor of TMT Number Sequencing scaled scores (higher scaled scores reflect faster completion times). The broken lines represent 95% prediction intervals.

HIP scores did not significantly predict TMT Condition 4 standard scores (see Table 2). Conversely, HIP scores significantly predicted TMT Condition 2 scores, even after accounting for age and education (*R*^2^ = 0.116, $F_{{\left( {3, 66} \right)}}$ = 2.875, *p* = 0.043), with a small-medium effect ($f^{2}$ = 0.131) and the HIP score as the sole significant predictor (HIP: *B* = − 0.235, CI [− 0.401, − 0.069], $t_{{\left( {66} \right)}}$ = − 2.830, *β* = − 0.331, semi-partial *r* = − 0.328, *p* = 0.006; age: *B* = − 0.005, CI [− 0.047, 0.036], $t_{{\left( {66} \right)}}$ = − 0.262, *β* = − 0.030, semi-partial *r* = − 0.030, *p* = 0.795; education: *B* = − 0.049, CI [− 0.135, 0.038], $t_{{\left( {66} \right)}}$ = − 1.128, *β* = − 0.132, semi-partial *r* = − 0.137, *p* = 0.264; see Fig. 5). Multicollinearity was not a concern for any of the predictors (all *Tolerance* values ≥ 0.981, all *VIF* values ≤ 1.019), and the data met assumptions of independent errors (*Durbin-Watson* = 2.10) and of non-zero variances (TMT Condition 2 *Variance* = 5.4). No other TMT conditions were significantly predicted by the model (see Table 2).

We further conducted posthoc analyses to test whether the change in performance between simple (i.e., conditions 1, 2, and 3) to complex (i.e., condition 4) attentional demands can be accounted for via hypnotizability. We computed delta values between the complex condition and each of the simple conditions. Our analyses revealed no significant relationship between the level of hypnotizability and the impact of increased cognitive demands on performance (see Table 2).

## Discussion

Our results indicate an inverse relationship between trait hypnotizability and perseveration, an executive function that utilizes regions of both the executive control and the salience systems. Consistent with our hypotheses, the hypnotizability-based prediction model and perseveration had significant yet relatively low (14.6%) shared variance. This finding can be interpreted as the representation of the shared mechanisms, while both perseveration and hypnotizability encapsulate more. Following the integrative model of hypnosis proposed by Lynn et al.^69^, we provide evidence that hypnotizability includes out-of-hypnosis cognitive processing characteristics that might influence responsiveness to suggestions in the hypnotic context. Conversely, based on our results, we rejected our hypotheses that higher hypnotizability will be associated with faster performance on simple attention tasks and with slower performance on more complex cognitive tasks. We also rejected the possibility that high hypnotizability is associated with a greater change in performance between the cognitive complexity conditions. Moreover, contrary to our hypothesis regarding simple attention performance, we observed a negative relationship between hypnotizability and performance on a basic psychomotor number sequencing task, whereby the more hypnotizable subjects were, the more slowly they completed the sequence. Although hypnotizability has been shown to relate to cerebellar control of sensorimotor integration^70^, it is unlikely that the observed relationship is due to a substantial motor factor as neither visual scanning nor motor speed (i.e., TMT conditions 1 and 5, respectively) was significantly related to hypnotizability. The observed relationship might represent a cognitive difference in the processing speed of number stimuli at different levels of hypnotizability. Neuroimaging and intracranial studies identified the intraparietal sulcus (IPS) as one of the major players in numerosity and numerical magnitude processing in the brain^71^. Cojan et al.^16^ reported differential activation of the IPS in relation to low versus high hypnotizability, particularly reduced recruitment of the IPS during selective attention tasks in highly hypnotizable individuals. It is possible that our finding corresponds to a relative reduction in the involvement of the IPS during the number sequencing task. However, due to the paucity of evidence to support such a hypothesis, we warrant caution when interpreting this result.

In their cold control theory of hypnosis, Dienes and Perner^72^ argued that higher levels of hypnotizability might relate to better executive control due to the tendency of high hypnotizables to suppress higher-order thoughts (i.e., the conscious awareness of intentions regarding the mental task or state) and, thus, their greater ability to assert more phenomenological control^73^. In the context of hypnosis, the cold control theory postulates that responsiveness to hypnotic suggestions involves intending to perform an action (which will lead to the success of the suggestions) while remaining metacognitively unaware of such intentions^73^. Dienes and Perner^72^ clarified that the tendency to be metacognitively unaware of intentions is not limited to hypnosis and, theoretically, should translate to any context when performance might feel as if it happens by itself. While better performance on the WCST is likely to involve intentional processes, the awareness of such intentions is not necessarily conditional for the successful completion of the task. Both empirical and theoretical evidence suggests that perseveration happens when a latent bias formed by previous experience outweighs a recent prefrontal-cortex-mediated active representation of a newly learned rule^74,75^. Put differently, to avoid perseverative responding, one must assert executive control to initiate a new rule while inhibiting the previous, automatically used cognitive paradigm. It is possible that greater awareness of intentions in performing on a task such as WCST increases the potential for dissonance between previous-experience bias and a novel alternative rule and, therefore, slightly increases the potential for perseveration. Insofar as high hypnotizables may be “better” at avoiding higher-order thinking, they might face less dissonance in abandoning the experience-based inapplicable logical rules and more easily accept alternative rules. In other words, high hypnotizables might feel as if the transitions between categories on the WCST or the strategy to do so “come up by themselves,” even though they are most likely generated through the same executive functions needed to perform the task in low hypnotizables. Such altered cognitive flexibility may indeed play a pivotal role in hypnotic responsiveness^69^, which supports Crawford and Gruzelier’s^5,25^ proposal that high hypnotizables have better cognitive flexibility outside the context of hypnosis.

Although our findings might appear inconsistent with Woody and Bowers’^76^ dissociated control theory (DCT), we argue that it does not. According to the DCT, highly hypnotizable individuals experience dissociation of the cognitive control system from the selective attention system during hypnosis^77^. Such dissociation was previously argued to resemble frontal lobe lesions^77^ and, as greater perseveration has been associated with impaired frontal lobe functions, linking less perseveration with high hypnotizability might conflict with predictions based on the DCT. However, our observations were made outside the context of hypnosis, while the dissociation argued for in the DCT happens within hypnosis and reflects a modality of cognitive control that is different from that which regulates cognitive performance outside the context of hypnosis^77^. Moreover, such dissociation has been theorized to manifest as dissociated connectivity between the ACC and PFC^77^, and recent evidence indicates a positive correlation between ACC-PFC connectivity and hypnotizability outside the context of hypnosis^6^. As some studies found no difference between hypnosis and non-hypnosis performance on more complex executive functions tasks of problem-solving and risk evaluation^78^, it is important to consider the possibility that frontal functions within- and outside hypnosis are not so different. Although our findings are not likely to represent dissociated connectivity between the ACC and PFC, they might represent altered recruitment of the PFC, outside hypnosis, in terms of reduced critical evaluative processing. The DCT argues that during hypnosis, inhibited frontally mediated functions in high hypnotizables lead to a diminished monitored execution of plans and strategies^76,77^. At this stage, Jamieson and Woody^77^ posited that external cues and communications become the main components that structure the content of cognitive processes, which reflects the heightened tendency to accept suggestions. This is, in fact, consistent with our hypotheses and findings insofar that high hypnotizables are more likely to accept an external alternative rule (e.g., corrective feedback on the WCST). Regardless of hypnotizability, participants’ performances on the WCST, including on measures of perseveration, were average. This suggests that, at least outside the context of hypnosis, higher hypnotizability might not manifest as frontal impairment, per se, but as alteration in frontal evaluative processes. It is also possible that frontal alterations in highly hypnotizables, as theorized in the DCT, do not substantially impact perseveration. Nevertheless, we do not believe our findings collide with the assumptions of the DCT and, if any, might be used to complement them.People often repeat irrelevant behaviors or thought patterns, despite recognizing the logical rules to which they should adhere^74^. When choice outcomes are consistent with the intended goals (e.g., matching feedback implies success), the frontal network is involved with the sense of agency with respect to the outcome^79^. However, this frontal involvement is absent when the outcomes do not match the goal (e.g., matching feedback implies erroring) but are not attributed to oneself^79^. This lack of attribution might also be enhanced by confirmation bias, an undervaluing of information that disconfirms previously held thought patterns, which has recently been associated with reduced neural sensitivity in the posterior portion of the medial PFC (mPFC) and can lead to inflexibility in processing novel or corrective information^80^. While perseveration is a rather common behavior, tests such as the WCST are designed to be sensitive to the pathological spectrum of cognitive functions. In the current study, most participants performed within the average range of cognitive performance, and the perseveration predicted by the HIP score should not be interpreted in terms of cognitive health, per se. The current study investigated the cognitive mechanisms underlying hypnotizability as a trait, and as expectations and preparatory responses play a bigger role within, rather than outside hypnosis^72,81^, the current findings might manifest differently within the context of hypnosis. The main limitation of our sample was a heavy-tailed and negatively skewed distribution of hypnotizability (see Table 1). Furthermore, although hypnotizability variances in our sample met the assumptions for non-zero variances, we did not have a balanced representation of high, medium, and low hypnotizable participants. For this reason, we chose to not perform comparisons of mean differences between high and low hypnotizability groups. As the original study^19^ recruited individuals with high and low scores on the HGSHS:A, we did not have neuropsychological performance data from the prospective participants who were excluded due to medium hypnotizability. Future research on the topic would benefit from having low, medium, and high hypnotizability groups with similar sample sizes. Moreover, including in-task neuroimaging data might help illuminate the relationships between trait hypnotizability and untimed executive control and information salience tasks. While this study is a step forward in our understanding of hypnotizability and its cognitive mechanisms, much is yet to be uncovered.
